# Attitudes of Elderly Population Toward Mobile Health Applications in Aseer Region, Saudi Arabia: A Cross-Sectional Study

**DOI:** 10.3390/healthcare13192464

**Published:** 2025-09-28

**Authors:** Nada Alqahtani, Dalia Almaghaslah

**Affiliations:** 1 Clinical Pharmacy Department, College of Pharmacy, King Khalid University, Abha 62521, Saudi Arabia; 2 Abha Maternity and Children Hospital (AMCH), Aseer Health Cluster, Abha 62523, Saudi Arabia

**Keywords:** mobile health applications, elderly population, digital health adoption, Saudi Arabia, usability barriers

## Abstract

**Background:** The rapid advancement of mobile health [mHealth] applications significantly improved healthcare accessibility. However, the adoption of these applications among the elderly population remains uncertain. This study aims to assess the attitudes of elderly individuals in the Aseer region, Saudi Arabia, toward mobile health applications, focusing on awareness, perceived benefits, usability concerns, and privacy issues. The findings will help guide strategies to enhance digital health adoption among older adults. **Methods:** A cross-sectional study was conducted among elderly individuals [≥60 years] residing in the Aseer region. A total of 500 participants were recruited using a non-probability convenient sampling technique. Data were collected through structured face-to-face interviews utilizing a validated questionnaire covering demographic characteristics, awareness, usage patterns, perceived benefits, and barriers to mHealth applications. Descriptive and inferential statistical analyses were performed using SPSS version 23.0. **Results:** The study included 500 elderly individuals in the Aseer region, Saudi Arabia. The majority of participants [70.4%] were aware of mobile health applications, with higher awareness among younger elderly [60–69 years, *p* = 0.008], individuals with higher income [*p* = 0.015], and those living with family [*p* < 0.001]. Mobile health apps were widely used, primarily for contacting healthcare providers [83.8%], managing appointments [79.4%], and medication management [79.2%]. Participants perceived these apps as highly useful, particularly for disease monitoring [mean = 4.34] and healthcare communication [mean = 4.34]. Awareness was negatively correlated with age [r = −0.109, *p* = 0.015], emphasizing the need for targeted educational interventions to enhance digital health adoption among older populations. **Conclusions:** Despite growing awareness, mHealth application usage remains limited among the elderly in the Aseer region due to digital literacy challenges and usability concerns. Addressing these barriers through user-friendly designs, targeted education, and privacy assurance measures may enhance adoption. Policymakers and healthcare providers should implement tailored interventions to promote digital health solutions for this population.

## 1. Introduction

### Background

There has been an increasing use of technology in healthcare worldwide, which highlights the importance of understanding the factors that influence its acceptance and use. There are several theories that explain technology acceptance, with the earliest and most influential being the technology acceptance model (TAM). TAM was developed to explain how perceived usefulness, perceived ease of use, and social norms influence the adoption of information systems. Since its introduction, the model has been extended and applied to a variety of technologies. Building on TAM, the unified theory of acceptance and use of technology (UTAUT) integrated eight different models and theories, including the theory of planned behavior, social cognitive theory, and the diffusion of innovations theory. A later refinement, UTAUT2, incorporated additional predictors of technology acceptance, such as hedonic motivation, price value, and habit. These models were largely developed and validated in the context of consumer adoption of information systems, with limited attention to their applicability for diverse technologies used by older adults [1].

The Saudi Vision 2030 aims to improve the quality of life, increase national economic development, and interact with the global community. One of its goals is to increase the use of mobile health [mHealth] apps in healthcare. Improved healthcare services and results via more accessible healthcare may be achieved through the integration of new technologies into the healthcare system [2,3,4]. The term “mHealth” refers to the use of mobile devices for remote communication in healthcare IT [5]. Now more than ever, mHealth forms the backbone of multiple successful applications across a wide range of healthcare domains, including as illness prediction, management, diagnosis, treatment planning, and patient education [6].

According to research from 2020, the app gives users the ability to interact with healthcare experts virtually, allowing them to receive healthcare from any location. Patients may also be able to see their lab results, request medicine refills, schedule appointments, and print out medical reports all via the mHealth app [7]. Using mHealth apps may increase healthcare service quality and overall satisfaction levels, according to research published the same year. The quick and simple medical treatment that consumers obtained was also mentioned. Reasons such as a lack of familiarity with, and training in the use of, modern mobile devices and their ever-evolving software systems contribute to the low rate of mHealth adoption in Saudi Arabia [8].

Recent studies in Saudi Arabia highlighted both the potential and the challenges of mHealth adoption among older adults. Meraya et al. [9] found that more than half of older adults with chronic conditions in the Jazan region used telehealth, with perceived usefulness and facilitating conditions strongly predicting positive attitudes, while barriers such as fear of technology and limited appointment availability reduced uptake. Similarly, Alzghaibi et al. [10] reported that non-healthcare users of the national *Sehaty* app identified technical issues, poor navigation, and privacy concerns as key obstacles that undermined satisfaction and engagement. Qualitative evidence from Almulhem et al. (2023) [11] further underscored that digital literacy, age-related limitations, and fear of mistakes hinder adoption, whereas family support, trust, and the perceived compatibility of apps with daily life facilitated use. Collectively, these studies suggest that while older adults in Saudi Arabia recognize the value of mHealth for managing health, sustained adoption depends on improving usability, addressing privacy concerns, and providing adequate training and support structures.

In the near and far future, public health outcomes may be improved by the use of mobile health [mHealth], particularly through the use of applications for smartphones, by focusing on issues such as chronic illness monitoring, medication adherence, etc., for instance [4]. There is evidence that even very easy treatments, such as text message reminders sent to a patient’s phone, may improve medication adherence and decrease appointment non-attendance [12,13]. While preliminary research on a variety of chronic diseases has shown promise for mHealth in illness management, there is a dearth of large-scale, long-term data on clinical results [14]. For instance, after 12 weeks, neither the intervention group nor the control group showed any significant change in blood pressure management in a recent randomized controlled study of an app for smartphones [15]. Whatever the case may be, mHealth is poised to take center stage in future public healthcare systems thanks to advancements in data analytics, AI, and smartphone technology.

The COVID-19 pandemic accelerated a global shift in healthcare delivery, emphasizing the necessity of remote and accessible services [16]. Restrictions on in-person visits, fear of viral transmission, and the pressure on healthcare systems made digital solutions, such as telehealth and mobile health [mHealth] applications, indispensable [17]. These technologies allowed patients to maintain continuity of care by consulting healthcare providers, monitoring chronic conditions, and accessing health information without physical contact [18,19]. As a result, reliance on telehealth not only persisted, but also strengthened in the post-pandemic era, supported by greater familiarity among patients and providers and increased investment in digital health infrastructure.

At the same time, the rapid development of smartphone technology enabled the creation of diverse health applications that can improve healthcare delivery and administration. However, cultural, technical, and age-related obstacles may affect attitudes toward these apps, particularly among the elderly, who often face challenges in adopting new technologies. This is especially evident in regions such as Aseer, Saudi Arabia, where demographic trends show a growing elderly population. In 2010, individuals aged 60 and above represented about 3% of Saudi Arabia’s population, a figure projected to rise to 9.5% by 2035 and 18.4% by 2050. Although region-specific data remain limited, evidence suggests a significant presence of elderly residents in Aseer.

The elderly have unique healthcare needs due to increased demand for medical care and continuous monitoring, making accessible and user-friendly digital health technologies particularly valuable. Yet, there remains a lack of data on their perspectives, intentions, and potential adoption drivers in this context. Understanding their awareness, perceived benefits, and barriers to use—as well as the demographic, cultural, and technological factors influencing their attitudes—is essential for designing effective, inclusive interventions.

Guided by H-TAM, we assess elderly adults’ attitudes toward smartphone health applications in Aseer and evaluate how perceived usefulness and facilitating conditions (age, income, and living arrangement), alongside health motivation (smoking status), relate to mHealth adoption and use.

## 2. Methods

### 2.1. Study Design

This study employed a cross-sectional design to explore elderly individuals’ awareness, use, and attitudes toward mobile health (mHealth) applications in the Aseer region of Saudi Arabia.

### 2.2. Study Setting

The research was conducted in healthcare centers and community locations across the Aseer region, where elderly individuals commonly access healthcare services or participate in community activities.

### 2.3. Population

The study population consisted of elderly individuals aged 60 years and above residing in the Aseer region (estimated N = 230,000).

### 2.4. Sample Size and Sampling Technique

The required sample size was calculated using the Epi-Info StatCalc module with a 95% confidence interval, 5% margin of error, and 50% expected frequency. This produced a minimum sample of 350 participants. To enhance representativeness and statistical power, 500 participants were recruited. A non-probability convenience sampling technique was employed to include participants from diverse geographic and socioeconomic backgrounds.

### 2.5. Mobile Applications

Mobile health applications in Saudi Arabia transformed healthcare accessibility, with prominent examples including *Sehhaty* and the National Guard Health Affairs (NGHA) application. *Sehhaty* provides appointment scheduling, medical record access, laboratory results, medication tracking, and health education resources. Similarly, the NGHA application allows appointment booking, prescription refills, and access to medical history. These applications demonstrate Saudi Arabia’s commitment to integrating digital technology into healthcare delivery.

### 2.6. Eligibility Criteria

Participants eligible for inclusion were elderly individuals aged 60 years and above, residing in the Aseer region, who owned or had access to a smartphone with e-health applications. Only those without cognitive, hearing, or speech impairments that could hinder their ability to complete the interview or provide informed consent were included. Exclusion criteria were elderly individuals with severe cognitive impairments or dementia, significant hearing or speech impairments preventing interview completion, or unwillingness to provide informed consent.

### 2.7. Data Collection Tool and Variables

The questionnaire was adapted from a previously validated instrument developed by Hossain et al. [20] and translated into Arabic using the standard back-translation method. It was culturally adapted to ensure clarity and relevance to the Saudi context, particularly for local mobile health applications. A pilot study with 20 older adults established face validity and confirmed clarity, suitability, and appropriateness.

The questionnaire included closed-ended and Likert scale questions. It captured demographic, lifestyle, and health information, along with awareness, usage, and attitudes toward mHealth. Items assessed sociodemographic details (age, gender, marital status, education, income, and living arrangements), lifestyle factors (smoking, weight, and height), and comorbidities. Participants were asked about mobile phone use for communication, time management, entertainment, financial transactions, and healthcare purposes. Specific mHealth functions, such as managing appointments, accessing records, tracking fitness, and contacting healthcare providers, were examined. Perceived usefulness of these functions and overall attitudes toward mHealth, including willingness to adopt and invest in such technologies, were also evaluated. Data were collected through structured, face-to-face interviews by trained research assistants. Given that interviews were face-to-face, confidentiality (not anonymity) was maintained. Identifiers were removed, and data were stored securely.

#### Application of H-TAM Framework

The healthcare technology acceptance model (H-TAM) informed the design of the questionnaire and guided the selection of study variables. Perceived usefulness was assessed through participants’ ratings of mHealth functions, while facilitating conditions were reflected in age, income, and living arrangements. Trust and health motivation were captured through smoking status, willingness to learn, and readiness to pay for mHealth. Embedding H-TAM ensured the study was conceptually grounded in an established framework for healthcare technology adoption.

### 2.8. Scientific Rigor

Instrument reliability was tested using Cronbach’s alpha, and content validity was reviewed by a panel of experts in gerontology and digital health. Pilot study results were used to refine the questionnaire for clarity and relevance.

### 2.9. Data Analysis

Quantitative data were analyzed using SPSS version 23.0. Descriptive statistics summarized demographic characteristics, awareness, usage, and attitudes. Inferential statistics, including chi-square tests and correlation analysis, were performed to examine associations between demographic factors and mHealth variables. Guided by H-TAM, the analysis specifically examined relationships between facilitating conditions (income, age, and living arrangements), health motivation (smoking status), perceived usefulness, and attitudes toward mHealth adoption.

Variable categorization: Perceived usefulness and attitudes were measured on 5-point Likert scales. Categories were created as follows:

- Low: mean score < 3.0
- Good: mean score 3.0–3.9
- High: mean score ≥ 4.0.

### 2.10. Ethical Consideration

Ethical approval was obtained from the Institutional Review Board at King Khalid University (Approval Number: ECM#20243-30130). All participants provided informed consent after being informed about the study’s purpose, procedures, and their right to withdraw at any time. Confidentiality of participants was strictly maintained throughout the study.

## 3. Results

### 3.1. Sociodemographic Characteristics

The study included 500 participants; just over half of them were males [n = 269, 53.8%]. The mean age among study participants was 68.18 ± 7.864 years and age ranged from 60 to 98 years. Most of study participants were married [n = 318, 63.6%]. In addition, less than a third of study participants had a university degree [n = 146, 29.9%]. Furthermore, most of study participants had good monthly income [n = 321, 64.2%]. A third of study participants had no previous employment [n = 168, 33.6%] and more than one third had been employed in governmental sector [n = 175, 35%]. Most of study participants are living with their families [n = 380, 76%]. The majority of study participants are non-smokers [n = 387, 77.4%]. Table 1 presents detailed sociodemographic characteristics of study participants.

### 3.2. Anthropometric Measurements

The mean weight among study participants was 69.91 ± 12.46 Kg with a median weight of 69 Kg. Weight ranged from 40 to 179 Kg. The mean height among study participants was 1.65 ± 0.11 m with a median height of 1.66 m. Height ranged from 1.10 to 1.91 m. The mean body mass index [BMI] among study participants was 25.76 ± 5.55 Kg/m^2^.

### 3.3. Comorbidity Status

**Figure 1:**
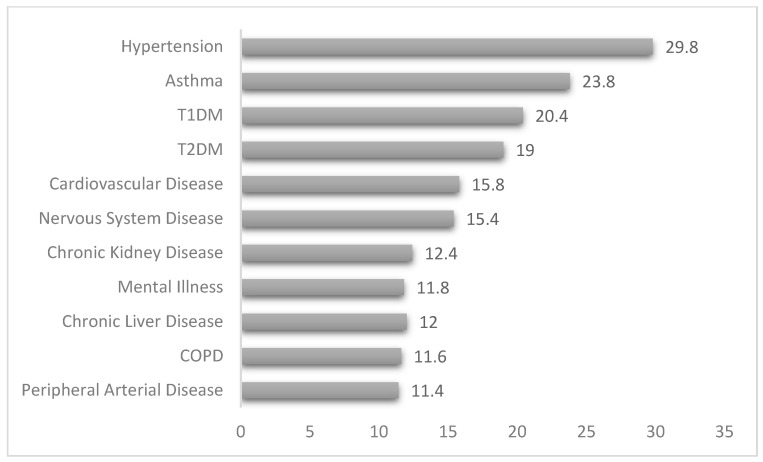
Comorbidity status among study participants.

Less than a third of study participants had a comorbid condition [n = 154, 30.8%] and some of them even had more than one comorbid condition. The most prevalent comorbid condition was hypertension [n = 149, 29.8%] followed by respiratory asthma [n = 119, 23.8%], as illustrated in Figure 1. Other comorbid conditions demonstrated include cardiovascular disease [CVD], peripheral arterial disease [PAD], chronic kidney disease [CKD], chronic liver disease [CLD], chronic obstructive pulmonary disease [COPD], and nervous system disease [NSD]. On the other hand, more than a third of study participants are using chronic medications [n = 199, 39.8%].

### 3.4. Usual Use of Mobile/Smart Phone

**Figure 2:**
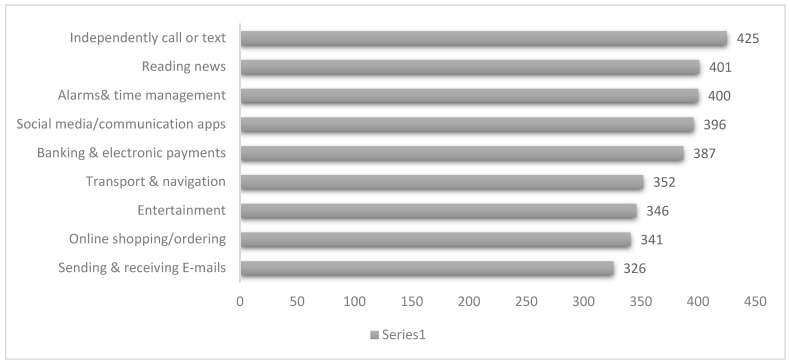
Mobile use purposes.

The study participants primarily used their mobile phones for various purposes, with the highest engagement observed in independent calling or texting [85.0%], followed by reading news [80.2%], alarms and time management [80.0%], and social media/communication apps [79.2%]. A significant proportion also utilized mobile phones for banking and electronic payments [77.4%], entertainment [69.2%], and online shopping or ordering [68.2%]. Sending and receiving emails was reported by 65.2% of participants. Transport and navigation were also commonly used features [70.4%]. These findings highlight the diverse functional roles of mobile devices in daily life, ranging from communication and organization to financial transactions and entertainment. Figure 2 shows frequency of mobile use purposes among study participants.

### 3.5. Awareness of Mobile Health Uses

The study participants demonstrated varying levels of awareness regarding mobile health applications, with the highest awareness reported for contacting healthcare providers [77.4%] and managing appointments [75.4%]. Awareness of medication management [73.8%] and accessing health information or education [72.4%] was also prevalent. Fitness and diet tracking [70.8%] and disease monitoring [70.6%] were similarly well-recognized. Meanwhile, awareness of accessing health records was relatively lower [66.6%]. Across all categories, a portion of participants were either unaware or uncertain about these mobile health features, highlighting the need for further education and promotion of digital health tools. Most of study participants were aware regarding mobile health application [n = 352, 70.4%]. Table 2 illustrates awareness level and relationship with sociodemographic characteristics.

There is a significant weak negative correlation between age and awareness score, as awareness increases when age decreases [r = −0.109, *p* = 0.015]. Statistically significant results indicate that awareness of mobile health varied significantly across age groups [*p* = 0.008], with higher awareness among those aged 60–69 years compared to older age groups. Income level was also a significant factor [*p* = 0.015], where individuals with good or high income showed greater awareness than those with weak income. Living alone was strongly associated with lower awareness [*p* < 0.001], as individuals living alone had significantly lower awareness than those living with others. Additionally, smoking status was a significant factor [*p* = 0.001], with non-smokers exhibiting higher awareness compared to ex-smokers and current smokers.

### 3.6. Actual Use of Mobile Health

**Figure 3:**
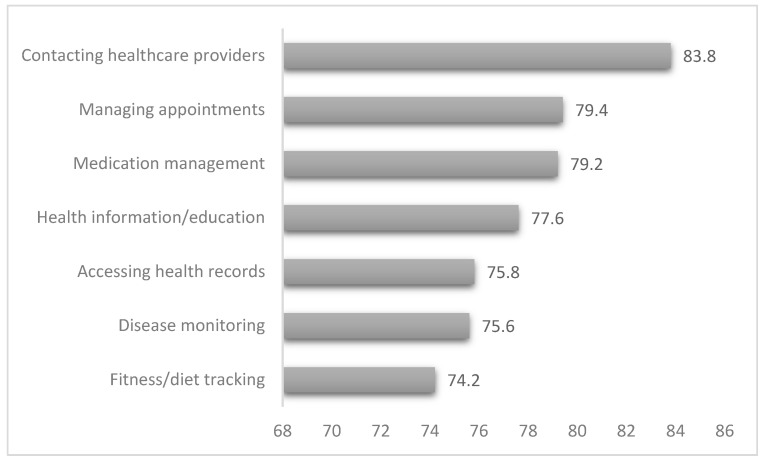
Actual use of mobile health and purposes.

The study findings indicate that mobile health applications were widely used among participants for various healthcare purposes. The most common use was contacting healthcare providers [83.8%], followed by managing appointments [79.4%] and medication management [79.2%]. Accessing health records [75.8%], obtaining health information or education [77.6%], and monitoring diseases [75.6%] were also prevalent. Additionally, fitness and diet tracking [74.2%] and medication management [79.2%] were frequently utilized. Figure 3 demonstrates previous findings.

### 3.7. Perceived Usefulness of Mobile Health

The participants’ responses to the perceived usefulness of mobile health applications indicate a strong positive perception across various functionalities. The highest-rated aspects were disease monitoring [mean = 4.34, SD = 1.187] and contacting healthcare providers [mean = 4.34, SD = 1.171], followed closely by medication management [mean = 4.32, SD = 1.234]. Fitness/diet tracking [mean = 4.28, SD = 1.239] and health information/education [mean = 4.28, SD = 1.216] were also highly rated. Managing appointments [mean = 4.15, SD = 1.350] and accessing health records [mean = 4.17, SD = 1.262] received slightly lower but still favorable ratings. The negative skewness in all items suggests that most participants perceived mobile health applications as highly useful, with the majority selecting “very useful” as their response. Table 3 presents detailed responses to scale items. Scale items had a good reliability [Cronbach’s Alpha = 0.930].

Statistically significant findings indicate that perceived usefulness of mobile health applications varied based on age group [*p* = 0.001], marital status [*p* < 0.001], income level [*p* = 0.002], living arrangement [*p* < 0.001], and smoking status [*p* < 0.001]. Participants aged 60–69 years had the highest proportion of individuals rating mobile health as highly useful. Married individuals reported higher perceived usefulness compared to other marital statuses. Those with higher income were more likely to perceive mobile health as useful. Additionally, participants who did not live alone and non-smokers had significantly higher perceived usefulness compared to those living alone and smokers. These results are presented in Table 4.

### 3.8. Attitude Toward Mobile Health

Participants generally exhibited a positive attitude toward mobile health, with mean scores above 4 for all items. The highest agreement was observed for willingness to learn about and try new mobile health solutions in the future [mean = 4.23, SD = 1.18], followed by willingness to pay for mobile health solutions [mean = 4.19, SD = 1.252] and the belief that mobile health has the potential to improve their health [mean = 4.16, SD = 1.318]. The negative skewness values indicate that responses were skewed toward agreement, suggesting an overall favorable perception of mobile health among participants. Further details are presented in Table 5. Scale reliability was good [Cronbach’s Alpha = 0.840]. The majority of participants had a positive attitude toward mobile health [n = 368, 73.6%].

Statistically significant findings indicate that age, income, living alone, and smoking status were associated with attitudes toward mobile health. Participants aged 60–69 years were more likely to have a positive attitude compared to older age groups [*p* < 0.001]. Those with higher income levels showed more positive attitudes toward mobile health than those with weaker financial status [*p* = 0.022]. Additionally, participants who did not live alone exhibited a more favorable attitude compared to those living alone [*p* = 0.010]. Smoking status also influenced attitudes, with non-smokers displaying a significantly more positive outlook on mobile health than current and ex-smokers [*p* = 0.010]. Further details are presented in Table 6.

## 4. Discussion

The findings of this study highlight a significant level of awareness and utilization of mobile health applications among elderly individuals in the Aseer region, with notable variations based on age, income, and living arrangements. The majority of participants recognized the value of these applications, particularly in facilitating communication with healthcare providers, managing medical appointments, and monitoring medications. However, awareness was significantly lower among older participants, suggesting potential barriers to digital health adoption in this subgroup. Within the healthcare technology acceptance model (H-TAM), these demographic and social differences can be understood as variations in facilitating conditions, where age-related challenges and limited resources constrain adoption. This underscores the need for targeted interventions aimed at increasing digital literacy and accessibility for older populations, particularly those aged 70 and above. These findings align with previous research indicating that younger elderly individuals are more likely to engage with digital health solutions, further emphasizing the role of socioeconomic and environmental factors in shaping technology adoption [16,21,22].

Older persons benefited from mHealth since it reduced caregiver stress, increased involvement among health professionals, and cut costs [23]. The use of mobile devices to assist medical and public health practices is known as mHealth [24]. However, there have been several obstacles encountered by the elderly when using mHealth, which might have a detrimental impact on their experience [18]. On top of that, the obstacles they encounter could be unique compared to those experienced by people of various ages. Several issues and hurdles have been highlighted in previous research, such as affordability, computer literacy, patients’ attitudes, physical and cognitive difficulties, and ease of use [25]. These map directly onto H-TAM constructs: affordability and literacy represent facilitating conditions, while usability reflects perceived ease of use. Barriers such as fear of mistakes and negative attitudes reflect challenges with trust, limiting sustained adoption.

A qualitative study among the Saudi elderly population explored factors influencing individuals’ intention to use mobile health (mHealth) applications in Saudi Arabia, guided by the healthcare technology acceptance model (H-TAM). Through semi-structured interviews with 14 elderly participants (aged 50+), three key themes emerged: factors affecting usage intention, concerns and barriers, and potential solutions. Key determinants included perceived usefulness, ease of use, trust, and family support, while major barriers involved digital illiteracy, health issues, fear of mistakes, and social concerns. Older individuals with lower education levels showed lower familiarity with mHealth. Addressing these challenges through user-friendly designs and targeted support can enhance mHealth adoption among the elderly [11]. Our findings complement this qualitative evidence by quantitatively demonstrating how perceived usefulness, facilitating conditions, and trust interact with demographics to influence adoption.

After controlling for participants’ smartphone use, those with jobs were more likely to utilize mHealth, which may reflect their greater disposable income. Employment status, in H-TAM terms, can be seen as a positive facilitating condition since it enhances both economic means and digital exposure. Our analysis also revealed that non-smokers reported more positive attitudes toward mHealth. Within the H-TAM framework, this can be explained through stronger health motivation: individuals who refrain from smoking are often more engaged in preventive health behaviors and thus more receptive to digital health tools that support disease management and wellness. Similarly, living with family was associated with greater willingness to adopt mHealth. In the Saudi cultural context, strong family ties and intergenerational support may provide both technical assistance and encouragement, acting as key facilitating conditions and sources of social influence that promote adoption. Our study participants were all Saudis, while some studies found differences among participants due to their ethnicity. There was an American study that found less minority participants in a diabetes mHealth program that encouraged patients to take their medication as prescribed by Nelson et al. [26]. However, Serrano et al. found that Hispanics were more open to exchanging mobile medicine reminders than non-Hispanic whites [27]. There needs to be further research on the impact of ethnicity on mHealth use. To better market their goods, application developers may also want to think about users’ diversity (for instance, by providing multilingual support).

Gender impact on mHealth adoption and use remains unknown. Consistent app usage was more common among female patients than male patients in the research by Goh et al. [28]. According to reports, female stroke patients were more open to the idea of using mHealth for blood pressure monitoring [29]. In contrast, app-assisted cancer treatment was more acceptable to German male oncology patients [30]. Still, we could not find any statistically significant variations between the sexes in our analysis. Gender disparities may be less pronounced in an older sample similar to ours, but H-TAM suggests that social influence and health motivation may explain some gender differences in adoption.

In contrast to our findings, Hossain et al. [20] reported that while mHealth use was low in Singapore, people generally had a favorable impression of it. The average usefulness score of mHealth was 0.7 out of 1, and 64 percent of people said they would be open to trying or learning more about it. Half of the people surveyed thought it may benefit their health Hossain et al. [20]. Surprisingly, views towards mHealth did not seem to be greatly impacted by age. Influence of age cannot be ignored, however, since several research have shown that the elderly have unfavorable views towards digital or mobile treatments [26,30,31]. Within H-TAM, this suggests that perceived usefulness and trust may be more critical predictors of adoption than chronological age alone.

In addition to showing favorable attitudes, a high percentage of participants were also prepared to pay for mHealth. This finding is reasonable given that most mHealth applications are currently offered free of charge to users [19]. More people were ready to pay for mHealth solutions if they lived in private, non-subsidized housing, which is a good indicator of socioeconomic level [20]. They were also more open to learning about or utilizing mHealth, and they were more inclined to agree that mHealth may enhance their health. Within H-TAM, willingness to pay can be interpreted as an expression of health motivation and trust in digital solutions. However, reliance on out-of-pocket payment may unintentionally exclude individuals with lower income or poorer health, underscoring the need for equitable financing models.

It is important to recognize the varying quality of existing mHealth applications in addition to demographic considerations and attitudes [32]. Some of these applications, especially those that help people remember to take their medications as prescribed, have been the subject of prior attempts at systematic evaluation. One restriction of the market at the time, as pointed out by Dayer et al., was that the majority of these applications were designed for consumers rather than healthcare professionals [33]. Though they did find a few high-quality applications that may be useful, Santo et al. discovered that most of them lacked desired characteristics [33]. Researchers are now looking at how well these applications work to improve adherence in individuals with coronary heart disease [34]. Within H-TAM, application quality is directly tied to perceived usefulness and trust—poorly designed apps undermine adoption, while integrated, reliable tools can enhance it. Healthcare professionals, including doctors, pharmacists, nurses, and allied health workers, should undertake active evaluation of such applications and consider their integration into practice.

## 5. Conclusions

This study underscores the increasing relevance of mobile health applications in Saudi Arabia, particularly in the Aseer region, where demographic shifts point to a growing elderly population with unique healthcare needs. The findings demonstrate that while awareness and usage of mHealth apps are relatively high, gaps remain among older, low-income, and socially isolated groups. Interpreted through the healthcare technology acceptance model (H-TAM), adoption is influenced by perceived usefulness, facilitating conditions, trust, and health motivation, highlighting the need to address barriers specific to the elderly.

National initiatives such as the Sehhaty and National Guard Health Affairs (NGHA) apps already represent major strides toward integrating digital health into daily medical care. However, their success among elderly populations depends on ensuring cultural appropriateness, user-friendly design, and strong privacy protections. Enhancing digital literacy programs, particularly in rural and underserved regions, will be vital for bridging the generational and technological divide.

By prioritizing inclusivity and tailoring mobile health solutions to the needs of Saudi elderly, policymakers and healthcare providers can strengthen the role of digital health in chronic disease management, preventive care, and patient empowerment. In doing so, Saudi Arabia can not only improve health outcomes for its aging population, but also set a regional benchmark for effective digital health adoption in line with Vision 2030 and its long-term healthcare transformation agenda.

## Figures and Tables

**Table 1:** Sociodemographic characteristics of study participants.

Characteristic		Frequency	Percent
Gender	Male	269	53.8
	Female	231	46.2
Age group	60–69 years	360	72
	70–79 years	84	16.8
	80–89 years	47	9.4
	90–99 years	9	1.8
Marital status	Single	50	10
	Married	318	63.6
	Divorced	44	8.8
	Widow	88	17.6
Education	Illiterate	132	26.4
	School	222	44.4
	University	146	29.2
Income	Weak < 5000 SAR/month	73	14.6
	Good 5000–10,000 SAR/month	321	64.2
	High > 10,000 SAR/month	106	21.2
Previous employment	None	168	33.6
	Governmental	175	35
	Private	64	12.8
	Personal/freelancer	76	15.2
	Others	17	3.4
Living alone	Yes	120	24
	No	380	76
Smoking Status	Non-smoker	387	77.4
	Ex-smoker	74	14.8
	Current smoker	39	7.8

**Table 2:** Awareness regarding mobile health among study participants.

Characteristic		Awareness Level		df	X^2^	*p* Value
		Aware	Not Aware			
Gender	Male	187	82	1	0.218	0.357
	Female	165	66			
Age group	60–69 years	266	94	3	11.748	0.008
	70–79 years	55	29			
	80–89 years	24	23			
	90–99 years	7	2			
Marital status	Single	32	18	3	6.954	0.073
	Married	235	83			
	Divorced	25	19			
	Widow	60	28			
Education	Illiterate	88	44	2	1.203	0.548
	School	159	63			
	University	105	41			
Income	Weak	41	32	2	8.400	0.015
	Good	235	86			
	High	76	30			
Previous employment	None	121	47	4	7.284	0.121
	Governmental	125	50			
	Private	45	19			
	Personal/freelancer	54	22			
	Others	7	10			
Living alone	Yes	66	54	1	17.970	<0.001
	No	286	94			
Smoking Status	Non-smoker	288	99	2	14.863	0.001
	Ex-smoker	39	35			
	Current smoker	25	14			

**Table 3:** Participants responses to perceived usefulness scale items.

Item	Response [Frequency]					Skewness	Mean	SD
	1	2	3	4	5			
Managing appointments	37	53	42	35	333	−1.258	4.15	1.350
Accessing health records	27	46	62	44	321	−1.251	4.17	1.262
Health information/education	33	22	55	53	337	−1.578	4.28	1.216
Fitness/diet tracking	34	28	44	50	344	−1.594	4.28	1.239
Disease monitoring	29	25	46	45	355	−1.709	4.34	1.187
Medication management	34	27	41	42	356	−1.669	4.32	1.234
Contacting healthcare providers	27	25	49	47	352	−1.693	4.34	1.171

1: Not useful at all; 2: a little bit useful; 3: sometimes useful; 4: usually useful; 5: very useful; and SD: standard deviation.

**Table 4:** Perceived usefulness regarding mobile health among study participants.

Characteristic		Perceived Usefulness			df	X^2^	*p* Value
		Low	Good	High			
Gender	Male	9	82	178	2	2.934	0.231
	Female	7	55	169			
Age group	60–69 years	11	83	266	6	22.914	0.001
	70–79 years	2	27	55			
	80–89 years	2	25	20			
	90–99 years	1	2	6			
Marital status	Single	2	13	35	6	29.040	<0.001
	Married	11	67	240			
	Divorced	2	24	18			
	Widow	1	33	54			
Education	Illiterate	6	35	91	4	4.154	0.386
	School	5	55	162			
	University	5	47	94			
Income	Weak	4	28	41	4	17.152	0.002
	Good	11	70	240			
	High	1	39	66			
Previous employment	None	4	41	123	8	5.649	0.686
	Governmental	7	50	118			
	Private	0	19	45			
	Personal/freelancer	4	22	50			
	Others	1	5	11			
Living alone	Yes	2	53	65	2	22.639	<0.001
	No	14	84	282			
Smoking Status	Non-smoker	12	88	287	4	20.186	<0.001
	Ex-smoker	3	30	41			
	Current smoker	1	19	19			

**Table 5:** Participants responses to attitude toward mobile health scale items.

Item	Response [Frequency]					Skewness	Mean	SD
	1	2	3	4	5			
I think mobile health has the potential to make me healthier.	38	42	46	50	324	−1.319	4.16	1.318
I am keen to learn about and try new mobile health solutions in future.	21	36	69	55	319	−1.336	4.23	1.18
I would be willing to pay for mobile health solutions.	37	20	70	55	318	−1.404	4.19	1.252

1: Strongly disagree; 2: disagree; 3: neutral; 4: agree; 5: strongly agree; and SD: standard deviation.

**Table 6:** Attitude toward mobile health among study participants.

Characteristic		Attitude			df	X^2^	*p* Value
		Negative	Neutral	Positive			
Gender	Male	17	57	195	2	1.019	0.601
	Female	10	48	173			
Age group	60–69 years	16	65	279	6	28.736	<0.001
	70–79 years	8	19	57			
	80–89 years	1	21	25			
	90–99 years	2	0	7			
Marital status	Single	2	9	39	6	6.083	0.414
	Married	14	67	237			
	Divorced	4	13	27			
	Widow	7	16	65			
Education	Illiterate	9	24	99	4	5.250	0.263
	School	13	42	167			
	University	5	39	102			
Income	Weak	8	17	48	4	11.395	0.022
	Good	18	60	243			
	High	1	28	77			
Previous employment	None	9	26	133	8	10.533	0.230
	Governmental	11	39	125			
	Private	1	21	42			
	Personal/freelancer	5	16	55			
	Others	1	3	13			
Living alone	Yes	6	37	77	2	9.226	0.010
	No	21	68	291			
Smoking Status	Non-smoker	20	68	299	4	13.319	0.010
	Ex-smoker	5	23	46			
	Current smoker	2	14	23			

## Data Availability

The original contributions presented in this study are included in the article. Further inquiries can be directed to the corresponding author.
